# An optimised liver-first strategy for synchronous metastatic rectal cancer leads to higher protocol completion and lower surgical morbidity

**DOI:** 10.1186/s12957-023-02946-6

**Published:** 2023-03-03

**Authors:** Julien Bonnet, Hélène Meillat, Jonathan Garnier, Serge Brunelle, Jacques Ewald, Anaïs Palen, Cécile de Chaisemartin, Olivier Turrini, Bernard Lelong

**Affiliations:** 1 Department of Digestive Surgical Oncology, Institut Paoli Calmettes, 232 Boulevard de Sainte Marguerite, 13009 Marseille, France; 2 Department of Radiology, Institut Paoli Calmettes, Marseille, France

**Keywords:** Rectal adenocarcinoma, Synchronous liver metastases, Liver-first, Rectal preservation

## Abstract

**Introduction:**

The optimal management of rectal cancer with synchronous liver metastases remains debatable. Thus, we propose an optimised liver-first (OLF) strategy that combines concomitant pelvic irradiation with hepatic management. This study aimed to evaluate the feasibility and oncological quality of the OLF strategy.

**Materials and methods:**

Patients underwent systemic neoadjuvant chemotherapy followed by preoperative radiotherapy. Liver resection was performed in one step (between radiotherapy and rectal surgery) or in two steps (before and after radiotherapy). The data were collected prospectively and analysed retrospectively as intent to treat.

**Results:**

Between 2008 and 2018, 24 patients underwent the OLF strategy. The rate of treatment completion was 87.5%. Three patients (12.5%) did not proceed to the planned second-stage liver and rectal surgery because of progressive disease. The postoperative mortality rate was 0%, and the overall morbidity rates after liver and rectal surgeries were 21% and 28.6%, respectively. Only two patients developed severe complications. Liver and rectal complete resection was performed in 100% and 84.6%, respectively. A rectal-sparing strategy was performed in 6 patients who underwent local excision (*n* = 4) or a watch and wait strategy (*n* = 2). Among patients who completed treatment, the median overall and disease-free survivals were 60 months (range 12–139 months) and 40 months (range 10–139 months), respectively. Eleven patients (47.6%) developed recurrence, among whom five underwent further treatment with curative intent.

**Conclusion:**

The OLF approach is feasible, relevant, and safe. Organ preservation was feasible for a quarter of patients and may be associated with reduced morbidity.

## Introduction

Better recommendations are needed for the surgical and oncologic management of synchronous rectal liver metastasis (LM) [1]. Despite oncologic progress [2], only complete resection (R0) or destruction of lesions at both sites allows medical remission [3]. The optimal strategy for the two surgical sites (i.e. the liver and rectum) is complex because it must consider many criteria, including treatment times and response to therapy. Management of rectal cancer involves long-course chemoradiation (CRT) potentiated by capecitabine followed by radical surgery with total mesorectal excision (TME) after a significant waiting period [4]. This treatment optimises regional control, but it is associated with a high morbidity and compromises subsequent management of liver metastasis if present in up to 50% of patients [5]. In patients with a good preoperative therapeutic response of the rectal tumour, a rectal-sparing strategy could reduce this morbidity, although this recommendation does not currently apply to patients with metastatic disease [6, 6, 7]. LM management requires systemic chemotherapy [8] and individualised surgical treatment ranging from staged hepatectomy to ablation. However, because these treatments must be tailored based on the response to chemotherapy [9], they can be time-consuming.

Regardless of the sequence, separate treatment programmes for the rectum and liver result in either a late start (rectum-first or classical strategy) or a prolonged interruption (liver-first or reverse strategy) of systemic chemotherapy during rectal management. The long cessation of chemotherapy puts patients at a high risk of liver disease progression and metastatic disease. Furthermore, up to 30% of these patients do not complete the planned liver and rectal resections because of disease progression [10].

Therefore, we proposed an optimised liver-first (OLF) strategy to reduce the time without systemic treatment and eventually the failure rate. In this strategy, all patients receive at least 6 cycles of induction chemotherapy followed by CRT. Resection of liver metastases is then performed within the recommended waiting period of 8 to 12 weeks between the end of CRT and rectal surgery, without delaying it.

The oncologic strategy is finally completed by adjuvant systemic chemotherapy. The objective of our study is to evaluate the feasibility and oncological quality of this OLF approach.

## Materials and methods

From January 2008 to January 2018, we evaluated all patients treated for low-mid rectal cancer (≤ 8 cm from the anal verge) with synchronous resectable or potentially resectable LM at the Institut Paoli Calmettes. Data were prospectively collected in a clinical database (NCT 02,869,503). The study protocol was conducted according to the 1989 World Medical Association Declaration of Helsinki and approved by our Institutional Review Board. The requirement for informed consent was waived (IPC 2020–041).

Pre-therapeutic evaluations included thoracoabdominal pelvic computed tomography (CT), endorectal ultrasonography (EUS), hepatic and rectal magnetic resonance imaging (MRI), and assessment of serum carcinoembryonic antigen (CEA). These tests were repeated at each step. Inclusion criteria were fitness for neoadjuvant treatment and surgery (performance status < 3) and expected margin-negative resection (R0) of the primary disease and LM. Patients with unresectable hepatic or extrahepatic disease or those treated with palliative treatment, previous pelvic radiotherapy, or emergency surgery were excluded. The case-by-case decision of the strategy was made based on the overall condition of the patient and determination of the resectability of the LM and rectal tumour based on multidisciplinary meetings.

### Medical treatment

All patients underwent neoadjuvant chemotherapy with platinum, fluorouracil, and/or irinotecan. The chemotherapy regimen was determined by the oncologist, surgeon, and patient based on the expected tumour response considering the recommendations and data in the literature [8, 11]. In line with these recommendations, patients received a total of 12 cycles. Adjuvant chemotherapy was performed after the rectal surgery in case of incomplete treatment.

In our department, preoperative CRT is indicated for patients with T3 and/or N + staged adenocarcinomas of the lower and middle rectum as well as ultra-low T2 tumours on the initial rectal MRI. Patients received preoperative normo-fractioned CRT (45–50 Gy in 25 fractions combined with capecitabine) [12]. Short-course radiotherapy was limited to patients with favourable lesions (small size with predictive circumferential margin (CRM) > 2 mm) or unfit patients [13].

### Liver management

The response to neoadjuvant chemotherapy was assessed 2–3 weeks after four to six cycles of chemotherapy using MRI and CT-based volumetric liver assessments. Portal venous embolisation (PVE) was performed to prevent postoperative liver failure if the expected future liver remnant was deemed to be less than 30% of the initial volume. Unresectable hepatic disease was defined by a consensus of liver surgeons taking into account the size, number, and unfavourable location of LM or a predicted insufficient future liver remnant. Liver surgery consisted of anatomical resection (segmentectomy or major or complex hepatectomy), non-anatomical tumorectomy, or thermoablation in one or two stages.

### Rectal management

The response to CRT was assessed 4–6 weeks after the last dose by EUS and pelvic MRI. Rectal surgery was performed 6 to 8 weeks after liver surgery and 8 to 12 weeks after the end of CRT. The type of surgery was defined according to the initial tumour characteristics and the therapeutic response [6, 7, 13]. The standard procedure was restorative TME. Patients with clinical persistent anal sphincter involvement or mrT4 tumours underwent abdominoperineal excision. For patients with complete or near-complete rectal clinical response (≤ mrT2 without node involvement), organ preservation was proposed, either full-thickness local excision (LE) [6] or a watch and wait strategy [7]. Completion TME was recommended at 1–4 weeks after LE for patients with a bad pathological response (ypT2-3 or R1).

### Histologic analysis

R0 liver resection was defined as microscopically tumour-free resection margins [14].

A rectal pathological examination was performed to assess distal and circumferential resection margins and the number of harvested lymph nodes. CRM involvement was defined as the presence of tumour cells ≤ 1 mm from the radial margin [15].

### *OLF strategy (Fig. **1**a, b)*

**Fig. 1:**
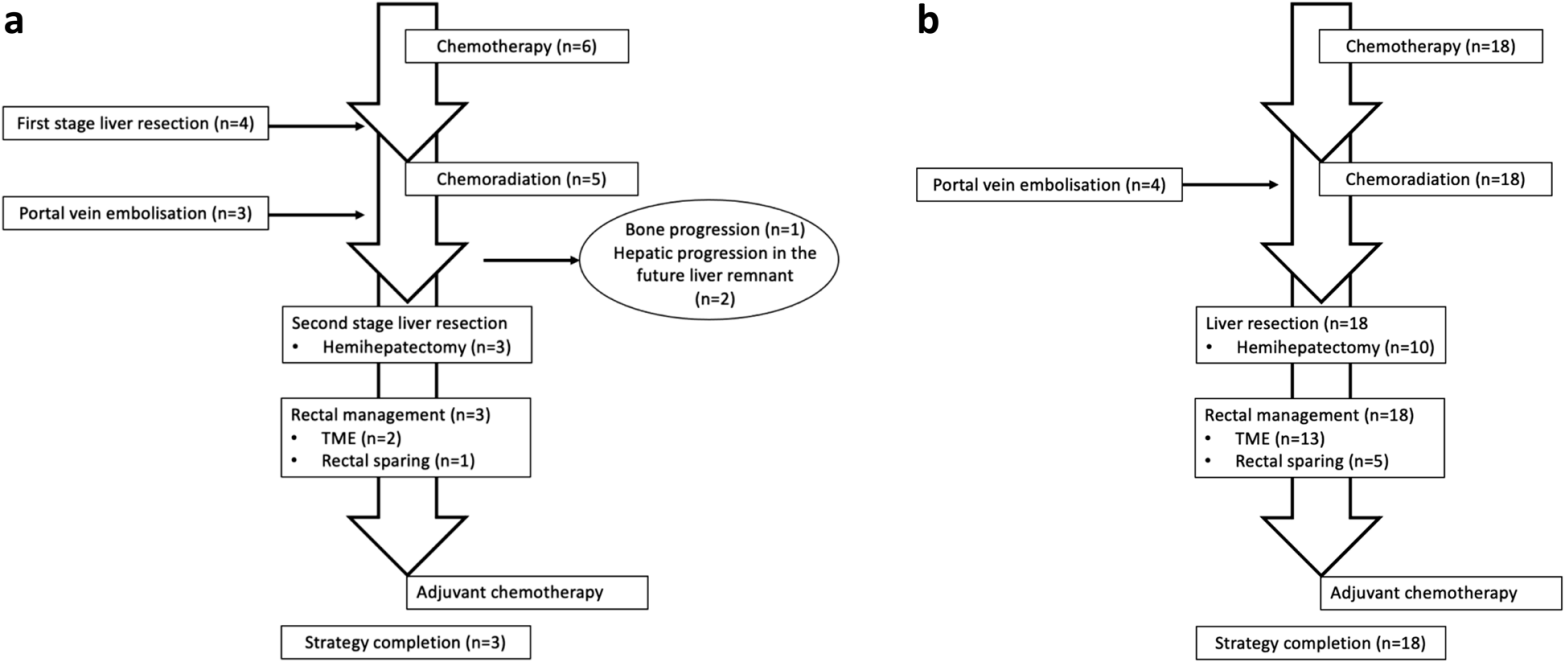
**a** Flow chart representing the scheme of the strategy in case of two-stages liver surgery. **b** Flow-chart representing the scheme of one-stage liver surgery

In this study, we examined an OLF strategy that combined concomitant pelvic irradiation with hepatic management. In patients undergoing one-step hepatic surgery, liver resection was performed in the interval between the end of pelvic CRT and the planned rectal surgery. In patients undergoing two-step hepatic surgery, the liver surgical procedures were performed before and after CRT, respectively; PVE was performed during CRT in some patients.

The choice of the strategy and the type of resection was left to the discretion of the surgeons and oncologists according to the post-therapeutic evaluation after confirming the absence of liver disease progression, adequate predicted volume of future liver remnant, and the rectal response.

### Outcomes

The feasibility of this approach was defined by the achievement of a curative strategy at both the hepatic and rectal sites, including rectal-sparing strategies and hepatic focal treatments. The oncologic quality was assessed by the R0 resection rate at both sites. Failure was defined by hepatic or extrahepatic progression or death.

### Postoperative management and follow-up

Postoperative morbidity and mortality were analysed at 90 days and classified using the Clavien-Dindo system [16]. Patients were followed up every 3 months for the first 2 years and then every 6 months for the next 3 years by clinical examination, thoracoabdominal CT scan, and serum CEA assessment. In addition, EUS and pelvic MRI were performed every 3 months in patients who underwent LE.

Local recurrence was defined as a radiologically and biopsy-proven tumour within the pelvis. Distant recurrence was defined as radiologic evidence of a tumour in any distant organ on imaging in the setting of elevated CEA. Overall survival (OS) and disease-free survival (DFS) were measured from the date of diagnosis. Patients considered to be disease-free were censored at the time of their latest clinical assessment follow-up. 

### Statistical analysis

Data analyses were performed using SPSS software (version 23; IBM Corporation, Armonk, NY, USA). Categorical variables were compared using Fisher’s exact test or the chi-squared test, and continuous variables were compared using Student’s *t*-test. The association of categorical variables with OS and DFS was assessed using the Kaplan–Meier method (based on the date of diagnosis and the relapse or censor date), and significance was tested using the log-rank test. Statistical significance was set at *p* < 0.05.

## Results

Between January 2008 and January 2018, 314 patients were treated for colorectal cancer with synchronous LM at our institution. Among them, 65 patients (20.7%) had rectal cancer, and 24 (7.6%) who underwent the OLF strategy were included. The characteristics of the study population are summarised in Table 1.

**Table 1:** Demographic and clinic data

	Overall cohort, no. (%)
Demographic data	
Sex	
—Male	18 (75%)
—Female	6 (25%)
Age, years^a^	64 (29–82)
BMI, kg/m^2^^a^	24.6 (18–37)
ASA score	
—1–2	17 (70.8%)
—3	7 (29.2%)
Pretreatment CEA level	
—< 200 g/L	15 (62.5%)
—≥ 200 g/L	5 (20.8%)
—Missing	4 (16.7%)
Rectal tumour	
Tumour location	
—Middle	11 (45.8%)
—Lower	13 (54.2%)
Pretreatment T stage	
—T3	22 (91.7%)
—T4a	2 (8.3%)
Pretreatment N stage	
—N0	4 (16.7%)
—N +	20 (83.3%)
Predictive threatened CRM (< 1 mm)	9 (37.5%)
Liver metastases	
Number of liver metastases^a^	2.5 (1–15)
> 3 lesions	9 (37.5%)
Size of the largest metastases^a^	40 (18–100)
> 5 cm	10 (41.7%)
Bilobar metastases	12 (50%)
Initial resectability	
—Resectable	19 (79.2%)
—Potentially resectable	5 (20.8%)
Preoperative treatment	
Chemotherapy regimen	
—Folfox	10 (41.6%)
—Folfirinox	11 (45.8%)
—Folfiri	3 (12.5%)
—Cetuximab/bevacizumab	6 (25%)
Radiotherapy modalities	
—Long course (50 Gy)	21 (91.3%)
—Short course (25 Gy)	2 (8.7%)

*ASA* American Society of Anesthesiologists, *BMI* Body mass index, *CEA* Carcinoembryonic antigen, *CRM* Circumferential resection margin

^a^Expressed as median (range)

### Oncological strategy

Twenty-one patients (87.5%) successfully underwent the OLF strategy and underwent both liver and rectal treatment (Fig. 1a, b). Three patients exited the strategy because of bone progression (*n* = 1) or unresectable hepatic progression (*n* = 2) after the first hepatic step. Three patients did not receive the planned chemotherapy after rectal surgery because of an altered general condition but were still considered to have completed the OLF strategy.

### *Liver surgery (Table **2**)*

**Table 2:** Surgical data and outcomes

Liver management (*n* = 24)	
Surgical procedure	
Single stage resection	18 (82.6%)
Including major hepatectomy	10/18 (55.5%)
Two-stage resection	6 (17.4%)
Including major hepatectomy	3/6 (50%)
Surgical approach	
Open	20 (83.3%)
Laparoscopic	4 (16.7%)
Conversion	0
Portal vein embolisation	8 (34.8%)
Postoperative complications	5 (20.8%)
Bile leakage	2 (8.3%)
Severe sepsis	1 (4.2%)
Clavien-Dindo classification	
Grade I–II	4 (80%)
Grade III–IV	1 (20%)
Margin status	
R0 resection	24 (100%)
Rectal management (*n* = 21)	
Surgical procedure	
Conservative TME	12 (57.1%)
Abdominoperineal excision	3 (14.3%)
Full-thickness local excision	4 (19.1%)
Watch and wait	2 (9.5%)
Abdominal surgical approach (*n* = 15)	
Laparoscopic	15 (100%)
Conversion	1 (6.7%)
Postoperative complications (*n* = 19)	6 (31.6%)
Anastomotic leakage	1/12 (8.3%)
Clavien-Dindo classification (*n* = 19)	
Grade I–II	5 (26.3%)
Grade III–IV	1 (5.3%)
Tumour classification (*n* = 19)	
ypT0	3 (15.8%)
ypT1	1 (5.3%)
ypT2	5 (26.3%)
ypT3	10 (52.6%)
Node classification (*n* = 15)	
ypN0	5 (33.3%)
ypN +	10 (66.7%)
Margin status (*n* = 19)	
R0 resection	16 (84.2%)
R1 resection	3 (15.8%)

Among the 24 patients who underwent liver surgery, major hepatectomy was planned in 15 patients and performed in 12 patients. PVE was necessary in 8 patients (33.3%). In patients undergoing one-stage liver surgery, the median interval between the last chemotherapy cycle and the liver surgery was 9 weeks (range 5–14 weeks). In patients undergoing two-step liver surgery, the median intervals between the last chemotherapy cycle and the first and second surgical steps were 4 weeks (range 3–6 weeks) and 11 weeks (range 9–17 weeks), respectively.

The postoperative mortality rate was 0%, and the overall morbidity rate was 21.7%. Only one patient had a severe complication and required radiological drainage of a biloma. The median length of stay (LOS) was 6 days (range 3–29 days). The R0 resection rate was 100%.

### *Rectal primary management (Table **2**)*

CRT and rectal management were performed in 21 patients (Fig. 1a, b). Six patients (28.6%) had a good or complete clinical response, and a rectal-sparing strategy was proposed, either watch and wait (*n* = 2) or LE (*n* = 4). TME was performed in the 15 remaining patients. The median interval between liver surgery and rectal surgery was 7 weeks (range 5–9 weeks).

The median interval between the end of CRT and rectal surgery was 10 weeks (range 7–16 weeks).

The postoperative mortality rate was 0%, and the overall morbidity rate was 28.6%, with only one severe complication (respiratory distress due to pneumopathy). The median LOS was 6 days (range 2–12 days). The R0 resection rate was 84.2% (16/19). Residual lymph node involvement was observed in 10 of 15 patients who underwent TME (66.7%). All patients who underwent a rectal-sparing strategy were initially classified as N1, but histopathological analysis confirmed a good tumour response, and no completion TME was necessary.

### Oncological outcomes

The median follow-up was 60 months (range 12–139 months). Local rectal recurrence occurred in two patients, one who underwent TME and one who underwent a watch and wait strategy, at 18 and 19 months, respectively. Eleven patients (42.8%) developed metastatic recurrence (liver only, *n* = 7; lung, *n* = 2; bone, *n* = 1; and peritoneal carcinomatosis,* n* = 1). Six (54.5%) patients underwent curative treatment for recurrence.

**Fig. 2:**
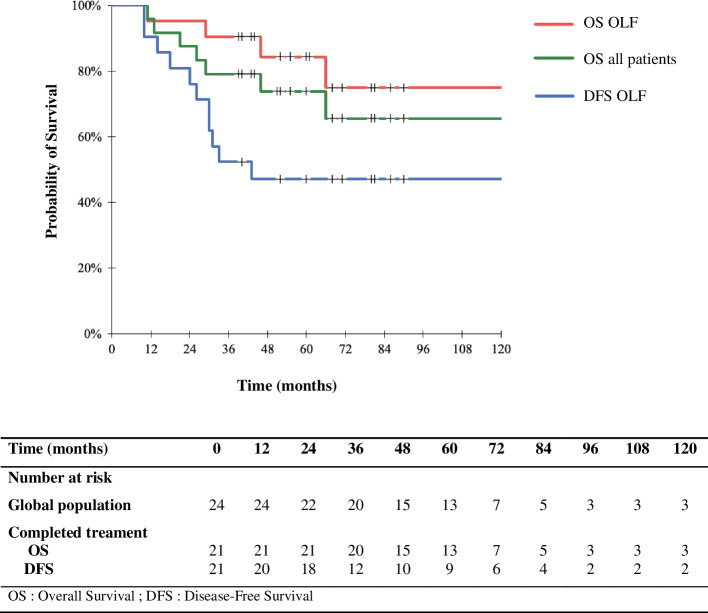
Survival curves

The median OS in all 24 patients was 57.5 months (range 12–139 months) (Fig. 2). The 1-, 3-, and 5-year OS rates were 100%, 83.3%, and 54.2%, respectively. The median OS and DFS in the 21 patients who completed the treatment were 60 months (range 12–139 months) and 40 months (range 10–139 months), respectively (Fig. 2). Among patients who completed the treatment, the 1-, 3-, and 5-year OS rates were 100%, 95.2%, and 61.9%, respectively. The 1-, 3-, and 5-year DFS rates were 95.2%, 57.1%, and 42.8%, respectively.

## Discussion

Our study suggests that the OLF strategy is an appealing approach in rectal cancer with synchronous LM. It was associated with a high completion rate, a low morbidity rate, and favourable long-term results. The liver-first strategy includes preoperative chemotherapy followed by resection of colorectal LM, with pelvic radiotherapy and resection of the primary bowel cancer in a second stage [17]. This strategy prioritises the removal of metastases without waiting for neoadjuvant therapy [17] or high infectious risk surgery of the primary rectal tumour [18]. However, this strategy still includes a chemotherapy-free period of at least 3 months after liver surgery. By combining the hepatic and rectal strategies, we propose resection of the LM during the interval between CRT and rectal cancer surgery. In the OLF strategy, the period without chemotherapy is shortened, which limits the risk of LM progression without compromising local control of the primary lesion.

Only 12.5% of our patients did not achieve the full strategy, all because of disease progression, which is comparable to what has been reported in previous studies examining delayed strategies of the rectal-first [19] or liver-first [20, 21] approaches. Some authors have shown that this ‘interval’ approach [22–24] with liver surgery performed between the end of CRT and rectal surgery has a comparable completion rate (89 to 100%). However, their protocols did not include routine preoperative chemotherapy and did not describe their postoperative chemotherapy protocol, which may possibly explain the higher recurrence rates than in our study. D’Hondt et al. described a 100% treatment completion rate, but they also had a hepatic recurrence rate of 55% and a median time to progression after liver surgery of 4.2 months [22].

The early application of systemic therapy allows for the treatment of metastatic disease by reducing the risk of LM progression while retaining local control of the rectal tumour [25]. The response to chemotherapy is a major prognostic factor in patients undergoing resection of colorectal LM [26]. Conversely, hepatic or extrahepatic progression during or even after the cessation of chemotherapy would contraindicate liver surgery or justify a change of chemotherapy [27]. Indeed, we consider the free interval during CR as a biological test for metastatic disease. In our study, three patients did not complete the treatment because of hepatic (*n* = 2) or extrahepatic (*n* = 1) progression at the systematic re-evaluation after 5 weeks of radiotherapy, i.e. within 8 weeks of the end of chemotherapy. According to Vigano et al., these patients have a particularly poor prognosis and will not benefit from liver surgery [27]. They also avoided unnecessary rectal surgery in the absence of symptomatic disease.

We therefore emphasise that our OLF strategy allows the achievement of long-course CRT without compromising systemic treatment or LM treatment and allows for patient selection.

Short-course radiotherapy (5 × 5 Gy) followed by consolidation chemotherapy [28] is a recently published alternative for locally advanced rectal tumours that could potentially be adapted for patients with metastases [29]. Although the oncologic outcomes of patients who underwent this strategy in a Dutch multicenter trial [29] were encouraging, the dropout rate was 35.2%, primarily due to hepatic progression. Therefore, we believe that it is legitimate to privilege an upfront chemotherapy.

On the other hand, the prioritisation of metastatic disease should not alter the management of the primary disease. Short-course radiotherapy would reduce the duration without chemotherapy but does not allow to consider a strategy of rectal sparing.

Only LE in patients with small T2T3N0-1 mid to low rectal cancer who have a good response to long-course CRT has been validated in a randomised controlled study [6]. Regardless of initial tumour stage, a complete histological response is associated with a good prognosis [30]. However, whether to maintain the indication for radical surgery in good responders has never been examined in patients with metastatic disease. Buchs and Mentha [31] reported a complete clinical response in three patients (9.7%), one of whom underwent LE; however, there was no information on his oncological follow-up. For Nierop, a rectal-sparing strategy could have been performed in 10 of 90 patients (11.1%) who underwent complete treatment [21]. In our study, a good therapeutic response after CT-CRT was observed in nine patients (42.8%), and six underwent a rectal-sparing strategy (28.5%). Only one patient, who underwent a ‘watch and wait’ strategy, developed hepatic recurrence 3 years after liver surgery and is currently undergoing chemotherapy.

The oncologic prognosis for rectal-sparing strategies is unknown in patients with metastatic disease, but it must be balanced with functional and morbidity benefits. Minimising operative morbidity is another important goal for treatment planning, as it is an independent factor of OS and DFS after colorectal LM resection [32]. We demonstrated that the OLF approach was associated with a very low overall morbidity rate; only two patients experienced a severe complication during treatment. In other studies evaluating the liver-first approach, the major morbidity rates varied from 0 to 33.3% [17, 23, 24] after rectal surgery and 0 to 27.3% after liver surgery [14, 22]. A randomised trial prospectively compared the morbidity between simultaneous and consecutive colorectal-first resections [19]. Severe morbidity rates were comparable between the 2 groups but very high compared to other studies. In the rectal cancer subgroup, the severe morbidity rates were 58.3% (7/12 patients) in the simultaneous group and 47.6% (10/21) in the delayed group. However, it is difficult to evaluate the direct impact of postoperative complications on the achievement of the second surgery in delayed strategies or on the administration of postoperative chemotherapy in the simultaneous approach. Only Conrad et al. detail this information; they found that 20% of the strategy’s failures were related to postoperative complications [33].

Although a high liver disease burden and locally advanced rectal cancer are common, we observed a high R0 resection rate at both sites compared with what has been previously described [19, 23, 31, 34]. Margin status is an important prognostic factor that may explain the high survival rate in our study compared with the results of other studies for rectal cancers with synchronous LM, regardless of the approach [19, 24] This favourable result could also be attributable to an optimisation of the management owing to the combination of neoadjuvant chemotherapy and preoperative radiotherapy with a limited chemotherapy-free interval.

The limitations of our study include the retrospective design and small sample size. Despite a long period of analysis, few patients benefited from this strategy. The choice of the OLF strategy depended on the initial disease characteristics and the inclusion period, with most of the patients being treated more recently. Additionally, this strategy requires coordinated action by a multidisciplinary team. Patients are not always referred to our centre upon diagnosis, which limits their potential inclusion and represents an obstacle to the generalised implementation of the OLF strategy. However, we included a recent and homogeneous population in an expert centre with a 5-year oncologic outcome assessment. Further, chemotherapy regimens and surgical indications remained stable throughout the study period.

## Conclusions

The present study suggests that the OLF strategy allows a greater selection of patients and an optimisation of the response at both sites. The optimal surgical procedure at each site can therefore be evaluated to ensure good oncologic resection with the lowest possible postoperative morbidity.

## Data Availability

The data that support the findings of this study are available from the corresponding author [HM], upon reasonable request. This manuscript has not been published or presented elsewhere in part or in entirety and is not under consideration by another journal.
